# Isolated Sensorineural Hearing Loss as a Sequela after Lightning Strike

**DOI:** 10.1155/2015/738416

**Published:** 2015-06-16

**Authors:** Mahfuz Turan, Ferhat Kalkan, Nazım Bozan, İsa Özçalimli, Mehmet Zeki Erdem, Abdülaziz Yalınkılıç, Mehmet Fatih Garca

**Affiliations:** ^1^Department of Otorhinolaryngology, Medical Faculty, Yüzüncü Yıl University, 65040 Van, Turkey; ^2^Department of Otolaryngology, Training and Research Hospital, Van, Turkey

## Abstract

In most of the surviving patients after a lightning strike, audiovestibular abnormalities have been reported. The most frequently reported type of abnormalities is a tympanic membrane perforation with hearing loss and external ear canal burn. However a sensor neural hearing loss and mixed type hearing loss can also occur, but these occur rarely. A nineteen-year-old female patient had, after a lightning strike, serious burns on the left ear, behind the ear, and on the chest and neck. She also had in her left ear 108 dB hearing loss with irregular central perforation and in her right ear 52 dB sensorineural hearing loss. There was no hearing loss before the strike. A hearing aid was recommended for the right ear and good care and follow-up were recommended for the left ear. A lightning strike can cause serious audiological damage. Therefore, it is necessary to make a careful audiovestibular evaluation of the patients. Although there exist rarely healed cases from sensorineural hearing loss after lightning strike in literature, in our case hearing loss occurred bilaterally and then it healed unilaterally. This condition is quite rare in literature.

## 1. Introduction

Lightning is a phenomenon in which an electric discharge of 40,000 to 50,000 A and temperature of 20,000°C are produced between the earth and the clouds, and it can cause important injury to humans [1]. Most of the fatalities from lightning strikes occur among young people who are engaged in outdoor activities [2].

Lightning strike has been reported as a sporadic cause of middle and inner ear damage [3]. Otologic injury from lightning has been sometimes notified, with perforation of the tympanic membrane (more than 50%) being the most common injury, along with ossicular disruption, vestibular organ injury with transient vertigo, tinnitus, and sensorineural hearing loss (50%) [4–6]. Even in cases of large tympanic membrane perforation temporal bone fractures and ossicular separation are not observed [7–9].

The pathophysiology of sensorineural hearing loss is described by several authors. This pathology may be attributed to vascular and structural changes. Firstly, hypoxia theory is defined as hypoxia in cochlear system secondary to the cardiorespiratory arrest [10]. Secondly, sympathetic variability and vasospasm may be another reason [11]. Additionally, disruption of inner ear anatomy, microhemorrhage, and microfracture, in the cochlea, may be responsible [4]. Audiovestibular sequelae after electrical injury (lightning and electric current) are often unannounced and are likely much more common than indicated in literature [2]. After lightning injuries, bilateral sensorineural deafness and positional vertigo emerged and died later owing to delayed transverse myelitis [9]. Transient vertigo is the most common vestibular symptom [2]. A case with progressive vertigo after lightning injury is reported which required labyrinthectomy 29 years after the initial injury [7]. The precise pathophysiology of vestibular lesions arising from lightning injury has not yet been fully understood and some theories are proposed. One of them is vascular damage that causes ischemia in the vestibular system. The other is changes in endolymphatic fluid system due to lightning injury. Another mechanism is thought that hypoxia occurs with decreasing arterial blood flow due to cardiac arrest after lightning injury [2].

External ear burns may occur [7, 12]. Although the tympanic membrane can be perforated, the ear canal may be intact [1, 8]. In this paper, we evaluated the cases like hearing loss, unilateral tympanic membrane perforation, and bilateral sensor neural hearing loss as a result of lightning strikes in the accompanying literature.

## 2. Case

**Figure 1:**
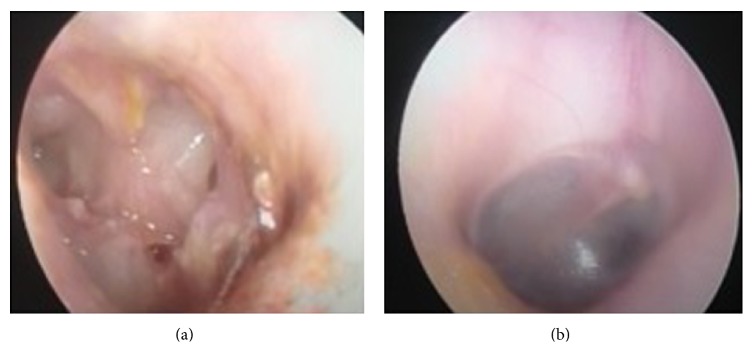
Left irregular perforated tympanic membrane plant (a) and right tympanic membrane intact (b).

**Figure 2:**
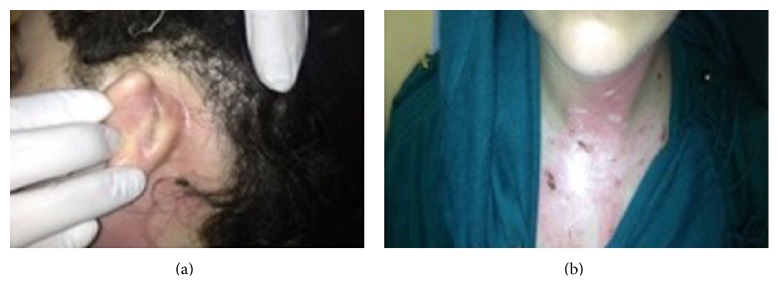
Skin burns in the postauricular, neck, and sternal region.

**Figure 3:**
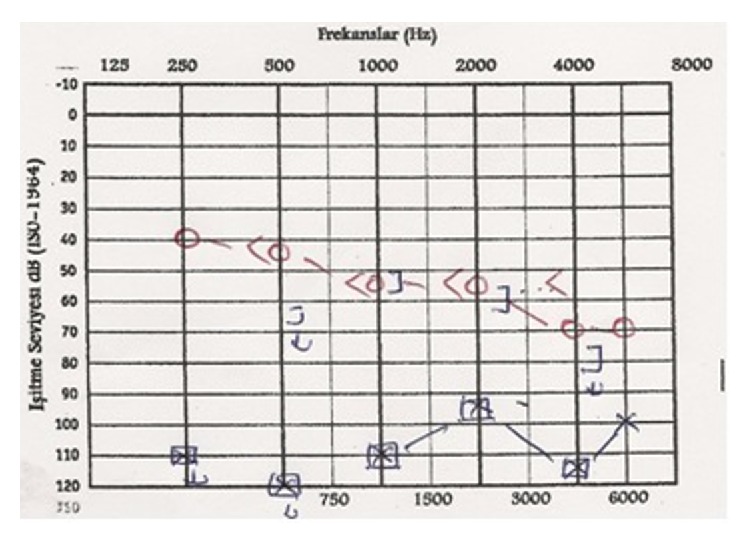
On the right ear 52 dB and on the left ear 108 dB sensorineural hearing loss.

**Figure 4:**
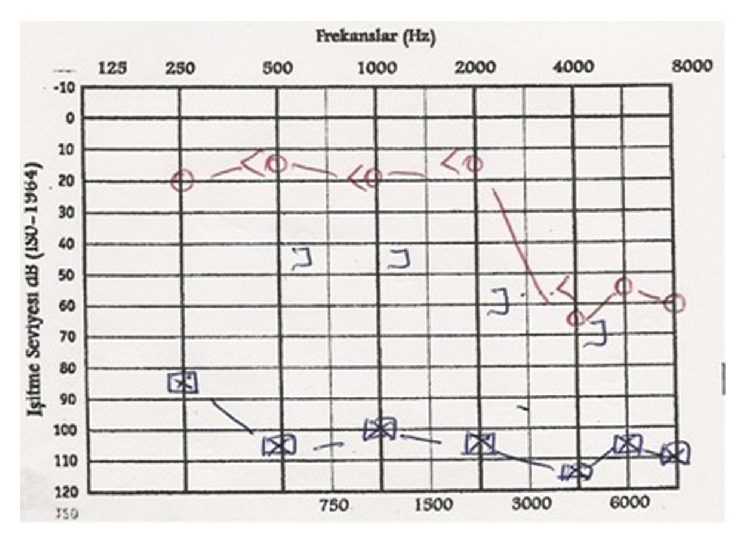
The degree of hearing loss in the right ear appears to show an improvement to the level of 17 dB.

A nineteen-year-old female patient, after being struck by lightning, came to the emergency room with burns on her body and somnolence. To observe the possible cardiac risks and for the treatment of burns on the body, the patient was treated in intensive care for eight days, and she applied to our otolaryngology clinic because of hearing loss ten days after lightning strike. It is written in her medical history that she had no hearing loss before the lightning strike and she cannot remember the case. During the physical inspection, right external auditory canal and tympanic membrane and the left external ear canal are examined as a normal. On the left tympanic membrane, about 5 × 4 mm perforation was determined (Figure 1(a)). Walk, Romberg, finger-nose, and Unterberger tests were found to be normal and there was no nystagmus; nasal and otorhinolaryngology examinations are determined to be normal. Healed burn wounds are observed on the posterior skin of the left ear, left side of the neck, and sternum (Figure 2). Based on these findings, we thought that lightning struck the left side of the body. During the pure tone audiometric on the left ear at 108 decibels (dB) and on the right ear 52 dB (average of hearing threshold in 500, 1000, and 2000 Hz frequencies) sensor neural hearing loss was determined (Figure 3). Magnetic resonance imaging of the cranial region was determined naturally. Additional pathology was not observed so, for the rehabilitation of the hearing, a hearing aid was given for the right ear. Protection principles were explained for left ear and regular follow-up was recommended. During the pure tone audiometric by the 2-year follow-up of the patient, the hearing loss on the right ear was 17 dB (average of hearing threshold in 500, 1000, and 2000 Hz frequencies) and there was no change in the severity of hearing loss in the left ear. On the other hand, hearing loss was continuing in high frequencies in audiogram (Figure 4). There were no changes on the size of the left tympanic membrane perforation. Patient did not accept the myringoplasty operation, which was recommended by us. Due to convalescence on her right ear, the use of hearing aids was terminated.

## 3. Discussion

Injuries from lightning may occur in four methods [1]. A direct strike is when a person absorbs the entire charge of the lightning strike as the energy passes through them. Aside flash happens when the lightning strike jumps from an object struck directly to a person standing nearby. Ground strikes happen when the lightening flash strikes the ground in close proximity to a person. Lightning also has an explosive effect and has the potential to bring about blunt injuries akin to those witnessed after an explosion [13]. The severity of the lightning strike varies depending on the duration of the shock, anatomic contact points of impact, and ways in which the current is passed. Anatomical point of contact of lightning with the body explains the possibility of direct tissue damage. The effect on tissues varies depending on the severity of impact during the lightning strike [7]. Despite the fixity of impedance of each tissue, the impedance of skin varies significantly depending on external factors and moisture content.

In most of the surviving patients after a lightning strike, audiovestibular abnormalities have been reported. The most frequently reported type of abnormalities is a tympanic membrane perforation with hearing loss and external ear canal burn. However a sensor neural hearing loss and mixed type hearing loss can also occur, but these occur rarely [12]. Wright and Silk have reported that in two cases of seven cases bilateral and in another four unilateral tympanic membrane perforations were observed and in one case tympanic membrane was healthy [7].

It is very difficult that tympanic membrane, middle ear, and labyrinth are affected by the current direction. It is because the impedance of these ways is higher than skin or soft tissue [8]. In our case, left tympanic membrane perforation and bilateral sensor neural hearing loss were developed. We think that this damage occurred due to proximity of the anatomical contact point to the audiovestibular system and also due to the duration of the shock during the lightning strike. It was not possible to determine the generation time of the lesion many years ago, because the audiometric measurements could not be done. However, when we accept major shock as a pathophysiological event, it can be thought that these types of cochlear pathology can occur depending on the acoustic trauma.

Variable factors affect the formation of inner ear lesions due to lightning strike. These are blast injury and the exposure of the direct cochlea throughout the electric current. Also the vessel rupture, which causes bleeding in the inner ear and endothelial damage, which causes occlusion by the small vessels, can be accused in this process as a result of long acting common vasospasm and sympathetic instability after a lightning strike. However, these mechanisms are estimates and may vary depending on the case. There are only a few temporal bone studies made on this topic. Particularly by the temporal bone studies of Youngs and friends on a patient who had bilateral sensor neural hearing loss and died because of transverse mellitus after a lightning strike, the absence of Corti member, rupture, and collapse by Reissner's membrane, strial degeneration, and reduction in the spiral ganglion cell populations were determined [9].

We thought that the reason for the hearing loss in our patient can be blast injuries or developed vasomotor change. The convalescence on the right ear of our patient can be explained with a long acting common vasospasm after lightening and improvement of endothelial damage, which causes occlusion in small vessels as a result of sympathetic instability and provision of revascularization. In our opinion, affection of the left side dominant hearing loss can result from the striking of the lightning to the left side of the body.

In the first-degree burns in auricle and external auditory canal daily dressing with cold water and analgesics is sufficient. In the second-degree burns, the burned area shall be cleaned with cold water and soap and then dead tissue shall be removed. The auricle shall be covered with antibiotic ointment and dressed with mastoid. There are full-thickness skin damage and probably tissue loss by third-degree burns so that cartilage can be seen. Skin grafts or local flaps are necessary for these patients. Oral and topical antibiotics should be used against secondary wound infections [12]. In our case, there was only a second-degree burn on the left outer ear canal. Daily aspiration and antibiotic ointment and dressings were applied to our patient.

Various animal experiments are done to explain the central and cochleovestibular pathology occurring due to lightning strike. Low voltage and alternating current have been used in animal models. However, these studies using animal models were insufficient to explain the size of the pathology as a result of lightning strikes, because the current shape and power of lightning strikes are variable [7].

In patients exposed to lightning strike, prognosis depends systemic effects, including primarily central nervous system. Renal failure due to muscle necrosis, myocardial dysfunction, and neurological disorders can occur after a lightning strike. The presence of developing neurological deficits is a serious condition and the most common of them is the loss of consciousness and confusion and these occur in 74–80% of all patients [14]. In our case, the patient had only amnesia during lightning period. Apart from that there were no past and recent amnesia and neurological deficits.

Tympanic membrane perforation and transmission type hearing loss due to lightning strike can heal by self-improvement of tympanic membrane or tympanoplasty. In the treatment of patients with tympanic membrane perforation due to lightning strike, except in cases with suspected fistula, spontaneous healing may be delayed due to local vascular structure and environmental damage. Therefore it is useful to wait up to six months for the tympanoplasty. If perforations do not heal within six months, the tympanoplasty is indicated [12]. In the three cases, reported by Redleaf and friends, myringoplasty was necessary for all of the perforations. But Jones et al., in only one case of 54 cases, myringoplasty was necessary and in the other cases perforation is healed spontaneously [12]. Also in one case with tympanic membrane perforation depending on the lightning strike, reported by Cankaya et al., perforation was healed by itself [15]. In our case, we have found that after 2 years of follow-up the perforation in the left ear still continued. The proposed myringoplasty operation was not accepted by patients.

## 4. Conclusion

Lightning strike can cause very different effects on auditory system. This may show different shapes like a simple change in hearing threshold up to total loss of hearing. It has to be considered that an audiovestibular pathology might occur the patients admitted to the emergency department after being struck by lightning and these patients have to be examined more carefully because of this reason. The convalescence on the right ear of our patient can be explained with a long acting common vasospasm after lightening and improvement of endothelial damage, which causes occlusion in small vessels as a result of sympathetic instability and provision of revascularization. But we think we need further investigations to clarify this situation.
